# Supplementary material for: Cellular stress alters 3′UTR landscape through alternative polyadenylation and isoform-specific degradation
Source: Nat Commun. 2018 Jun 11;9:2268. doi: 10.1038/s41467-018-04730-7 (PMC5995920; doi:10.1038/s41467-018-04730-7)
Supplement: Supplementary file 1 — Supplementary Information [file 41467_2018_4730_MOESM1_ESM.pdf]

Supplementary Information

Cellular stress alters 3'UTR landscape through alternative polyadenylation and isoform-specific degradation

Dinghai Zheng[1, 2, 4], Ruijia Wang[1, 2, 4], Qingbao Ding[1, 2], Tianying Wang[3], Bingning Xie[1, 2], Lu Wei[1, 2], Zhaohua Zhong[3], Bin Tian[1, 2, *]

[1]Department of Microbiology, Biochemistry and Molecular Genetics, Rutgers New Jersey Medical School, Newark, NJ 07103, USA
[2]Rutgers Cancer Institute of New Jersey, Newark, NJ 07103, USA
[3]Department of Microbiology, Harbin Medical University, Harbin 150081, China

[4]These authors contributed equally to this work.

[*]**Corresponding author:**

Bin Tian
E-MAIL: btian@rutgers.edu
TEL: (973) 972-3615
FAX: (973) 972-5594

Supplementary Tables

Supplementary Table 1. RT-qPCR primers used in this study.

Gene Name	Targeting Region	Direction	Sequence
Calm1	Common	Forward	CCTAGTGAACCATGACTCGGA
Calm1	Common	Reverse	AACAGCAATGTTGATGGTGTG
Calm1	aUTR	Forward	TGACAAAGAGTTGTACTTGGACG
Calm1	aUTR	Reverse	TTCCATAGAGTTGGTCCCCC
Dnajb1	Common	Forward	CACCTGAGAAACGTGGAGACC
Dnajb1	Common	Reverse	GTGCAGGTGGCTATATGGGA
Dnajb1	aUTR	Forward	ACTCAGACGAAAGCAGTCCC
Dnajb1	aUTR	Reverse	CACCAGGCTAGCTCCAAAGAA
Fam49b	Common	Forward	CTGCATGGCGAAACTGCCTA
Fam49b	Common	Reverse	TCCATTTTGGCTGCACATGA
Fam49b	aUTR	Forward	GTAATCCTGCTGTGGCCTTTC
Fam49b	aUTR	Reverse	CACCGACTGGGCTACTGAC
Hspa4l	Common	Forward	GCCTATGGGGAAGCACTGTT
Hspa4l	Common	Reverse	TTGTGCAACGTCTCCCTACC
Hspa4l	aUTR	Forward	TGCAAAGCAGTGCAGAAAACT
Hspa4l	aUTR	Reverse	TGGAAGCCGTTCTTATGTCTGT
Nmt1	Common	Forward	GCTGCGTGATGTCACCTTTA
Nmt1	Common	Reverse	TCACGAGCTAGGTTTCAGCA
Nmt1	aUTR	Forward	ATCTGAGGGTCGGAGAGCTT
Nmt1	aUTR	Reverse	ATTGGGTATCCCAGCAATGA
Purb	Common	Forward	AGCCCTCCAGGCCTCACTAC
Purb	Common	Reverse	TTGCCCTGTCCATGATTGTG
Purb	aUTR	Forward	AATCCCACCCCATCAAAAGG
Purb	aUTR	Reverse	AGGGCAGGCATGCAGTTAAA
Rpl22	Common	Forward	GGAGTCGTGACCATCGAACG
Rpl22	Common	Reverse	GCCAGTCTCGGAGGTTGTTC
Rpl22	aUTR	Forward	GCGTTTCCTTCCAACTTCAGG
Rpl22	aUTR	Reverse	GGTCCGGCGATGTAACAAAA
Timp2	Common	Forward	TTTCTTGACATCGAGGACCC
Timp2	Common	Reverse	TCCAGGAAGGGATGTCAAAG
Timp2	aUTR	Forward	ATGTGCGTGCTGGAATATGA
Timp2	aUTR	Reverse	CTGATACAGAGCATCAGGCG

Supplementary Table 2. Sequencing samples used in this study

Sample ID	Sequencing method	Raw read number	Sample description[1]	SRA accession ID
1	RNA-seq	31,145,949	Total RNA, NIH3T3 cells, NT, replicate 1	SRX3033634
2	RNA-seq	35,187,433	Total RNA, NIH3T3 cells, AS, replicate 1	SRX3033635
3	RNA-seq	27,629,537	Total RNA, NIH3T3 cells, NT, replicate 2	SRX3033636
4	RNA-seq	35,600,384	Total RNA, NIH3T3 cells, RC, replicate 1	SRX3033637
5	3'READS	16,752,923	Total RNA, NIH3T3 cells, NT, replicate 1	SRX3033649
6	3'READS	10,309,635	Total RNA, NIH3T3 cells, AS, replicate 1	SRX3033650
7	3'READS	18,362,539	Total RNA, NIH3T3 cells, NT, replicate 2	SRX3033651
8	3'READS	17,595,096	Total RNA, NIH3T3 cells, AS, replicate 2	SRX3033652
9	3'READS	35,894,455	Total RNA, NIH3T3 cells, AS, replicate 3	SRX3033653
10	3'READS	43,239,461	Total RNA, NIH3T3 cells, RC 12 h, replicate 1	SRX3033654
11	3'READS	36,032,739	Total RNA, NIH3T3 cells, RC 24 h, replicate 1	SRX3033655
12	3'READS	45,805,336	Total RNA, NIH3T3 cells, RC 4 h, replicate 3,	SRX3033656
13	3'READS	44,947,807	Total RNA, NIH3T3 cells, RC 8 h, replicate 1	SRX3033657
14	3'READS	41,243,354	Total RNA, NIH3T3 cells, NT, replicate 3	SRX3033658
15	3'READS	37,619,965	4sU-labeled RNA, NIH3T3 cells, NT, replicate 1	SRX3033667
16	3'READS	34,322,197	4sU-labeled RNA, NIH3T3 cells, AS, replicate 1	SRX3033668
17	3'READS	53,111,239	4sU-labeled RNA, NIH3T3 cells, NT, replicate 2	SRX3033669
18	3'READS	56,122,798	4sU-labeled RNA, NIH3T3 cells, RC, replicate 1	SRX3033670
19	3'READS	30,025,438	Flow-through RNA, NIH3T3 cells, NT, replicate 1	SRX3033671
20	3'READS	28,587,835	Flow-through RNA, NIH3T3 cells, AS, replicate 1	SRX3033672
21	3'READS	34,054,203	Flow-through RNA, NIH3T3 cells, NT, replicate 2	SRX3033673
22	3'READS	32,468,941	Flow-through RNA, NIH3T3 cells, RC, replicate 1	SRX3033674
23	3'READS	9,860,263	RIP input RNA, NIH3T3 cells, NT, replicate 1	SRX3465843
24	3'READS	8,843,709	RIP input RNA, NIH3T3 cells, NT, replicate 2	SRX3465844
25	3'READS	9,502,287	RIP input RNA, NIH3T3 cells, AS, replicate 1	SRX3465841
26	3'READS	6,833,382	RIP input RNA, NIH3T3 cells, AS, replicate 2	SRX3465842
27	3'READS	11,915,192	TIA1 RIP RNA, NIH3T3 cells, NT, replicate 1	SRX3465847
28	3'READS	18,573,564	TIA1 RIP RNA, NIH3T3 cells, NT, replicate 2	SRX3465848
29	3'READS	12,820,233	TIA1 RIP RNA, NIH3T3 cells, AS, replicate 1	SRX3465845
30	3'READS	13,055,633	TIA1 RIP RNA, NIH3T3 cells, AS, replicate 2	SRX3465846
31	3'READS	14,684,835	Cytoplasmic RNA, proliferating C2C12 cells, NT, replicate 1	SRX3033675
32	3'READS	10,662,954	Cytoplasmic RNA, proliferating C2C12 cells, NT, replicate 2	SRX3033676

33	3'READS	19,527,790	Cytoplasmic RNA, proliferating C2C12 cells, AS, replicate 1	SRX3033677
34	3'READS	19,684,160	Cytoplasmic RNA, proliferating C2C12 cells, RC, replicate 1	SRX3033678
35	3'READS	18,959,803	Cytoplasmic RNA, differentiated C2C12 cells, NT, replicate 1	SRX3033679
36	3'READS	19,540,699	Cytoplasmic RNA, differentiated C2C12 cells, NT, replicate 2	SRX3033680
37	3'READS	16,298,332	Cytoplasmic RNA, differentiated C2C12 cells, AS, replicate 1	SRX3033681
38	3'READS	17,737,491	Cytoplasmic RNA, differentiated C2C12 cells, RC, replicate 1	SRX3033682
39	3'READS	12,901,771	Total RNA, differentiated C2C12 cells, replicate 1	SRX3033683
40	3'READS	16,763,626	Total RNA, differentiated C2C12 cells, replicate 2	SRX3033684
41	3'READS	11,280,672	Total RNA, proliferating C2C12 cells, replicate 1	SRX3033685
42	3'READS	16,568,113	Total RNA, proliferating C2C12 cells, replicate 2	SRX3033686

[1]RC samples are 4 h of recovery unless otherwise indicated.

Supplementary Table 3. Cloning primers used in this study

Gene, Region	PCR Primers
Nmt1, 3'UTR	5'-ATGCATGCCTCGAGCCAGTTGCCAGTGAGATTCTG;<br>5'-ATGCATGCGCGGCCGCGACCGTTGAACTTCCTTTATTAGAAACA
Nmt1, cUTR	5'-ATGCATGCCTCGAGCCAGTTGCCAGTGAGATTCTG;<br>5'-ATGCATGCGCGGCCGCATATATAATATATATACTTTTATTATTCACCCGTTAA CTCCATAATATG
Dnajb1, 3'UTR	5'-ATGCATGCCTCGAGCCACCTGCACTCCTCAAGGA;<br>5'-ATGCATGCGCGGCCGCTGAGGTTTAGCATCAGTCTTTAATGCTG
Timp2, 3'UTR	5'-ATATATCTCGAGGAAGGCTGACAGAGCCCCT;<br>5'-ATATATGCGGCCGCCAACTGAGGCACACCTTCAG

Supplementary Figure 1

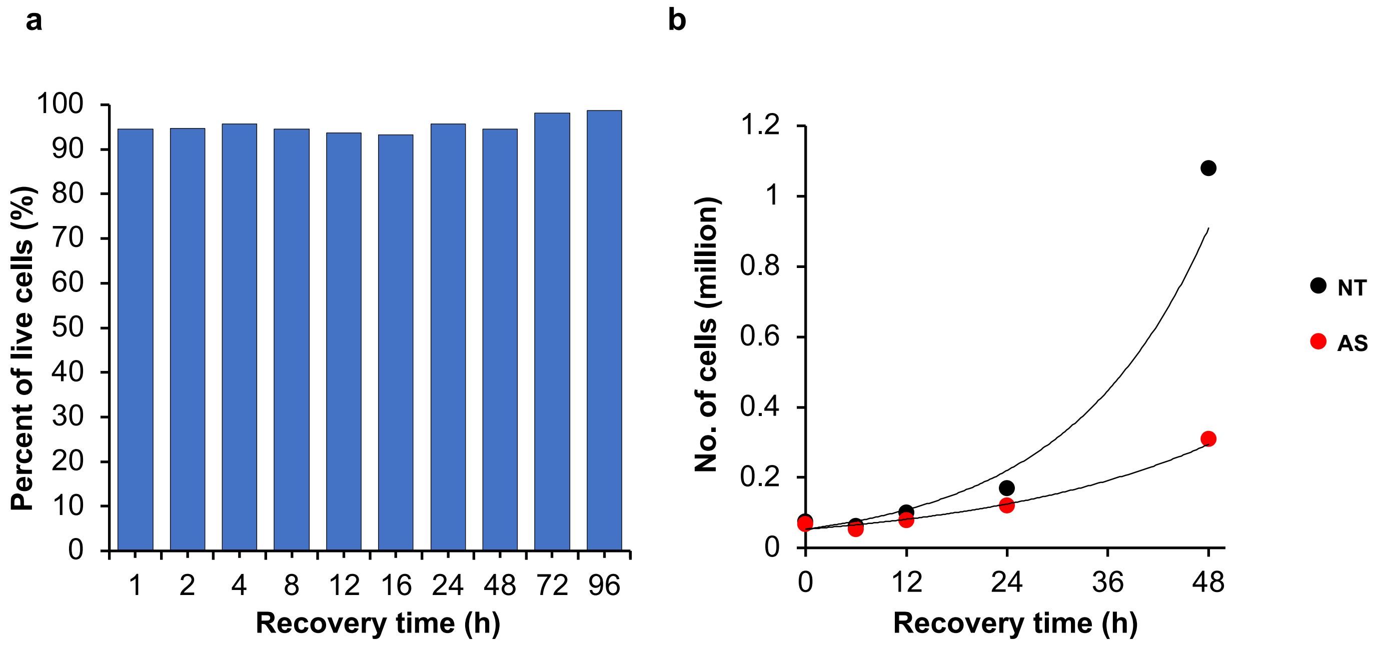

Supplementary Figure 1. Cell survival and growth analyses after 1 h of 250 µM sodium arsenite (AS) treatment. (a) Percentage of live cells measured by Trypan blue staining at different time points during recovery from stress. **(b)** Cell growth after AS treatment. Growth curves were fitted with an exponential lines.

Supplementary Figure 2

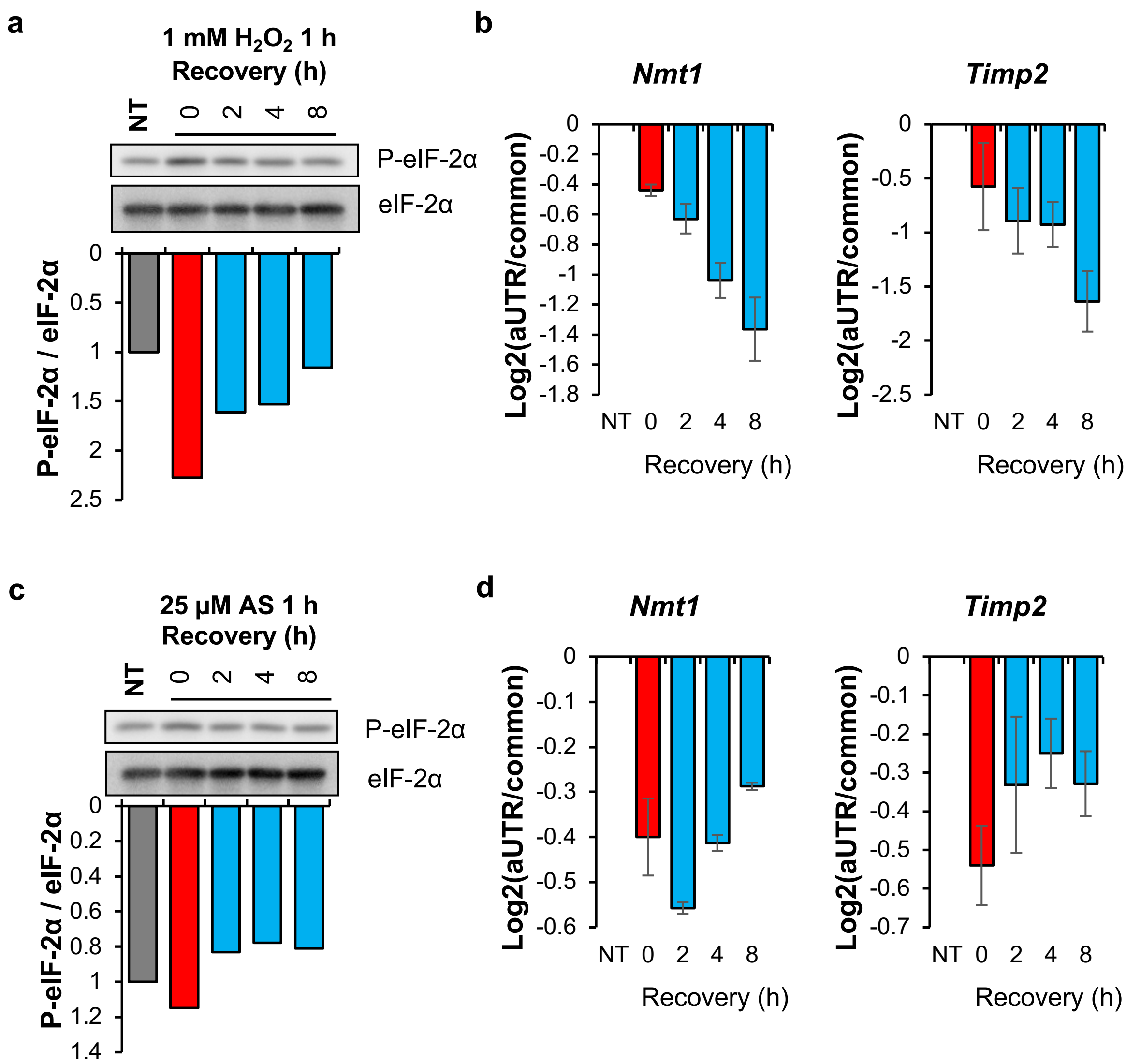

Supplementary Figure 2. APA analysis of cells under different types of stresses. (a) Top, Western blot analysis of phosphorylated (upper) and total (lower) eIF-2α protein in cells without treatment (NT) or at indicated time points after 1 h of 1 mM H_2O_2 treatment. Recovery time points are indicated. NT, no treatment. Bottom, normalized ratio of amount of phosphorylated eIF-2α to that of total based on the Western blot data. **(b)** RT-qPCR analysis of APA of example genes in cells without treatment (NT) or at indicated time points after 1 h of 1 mM H_2O_2 treatment. Error bar: s.d. **(c)** Top, Western blot analysis of phosphorylated (upper) and total (lower) eIF-2α protein in cells without treatment (NT) or at indicated time points after 1 h of 25 μM AS treatment. Recovery time points are indicated. NT, no treatment. Bottom, normalized ratio of amount of phosphorylated eIF-2α to that of total based on the Western blot data. **(d)** RT-qPCR analysis of APA of example genes in cells without treatment (NT) or at indicated time points after 1 h of 25 μM AS treatment. Error bar: s.d.

Supplementary Figure 3

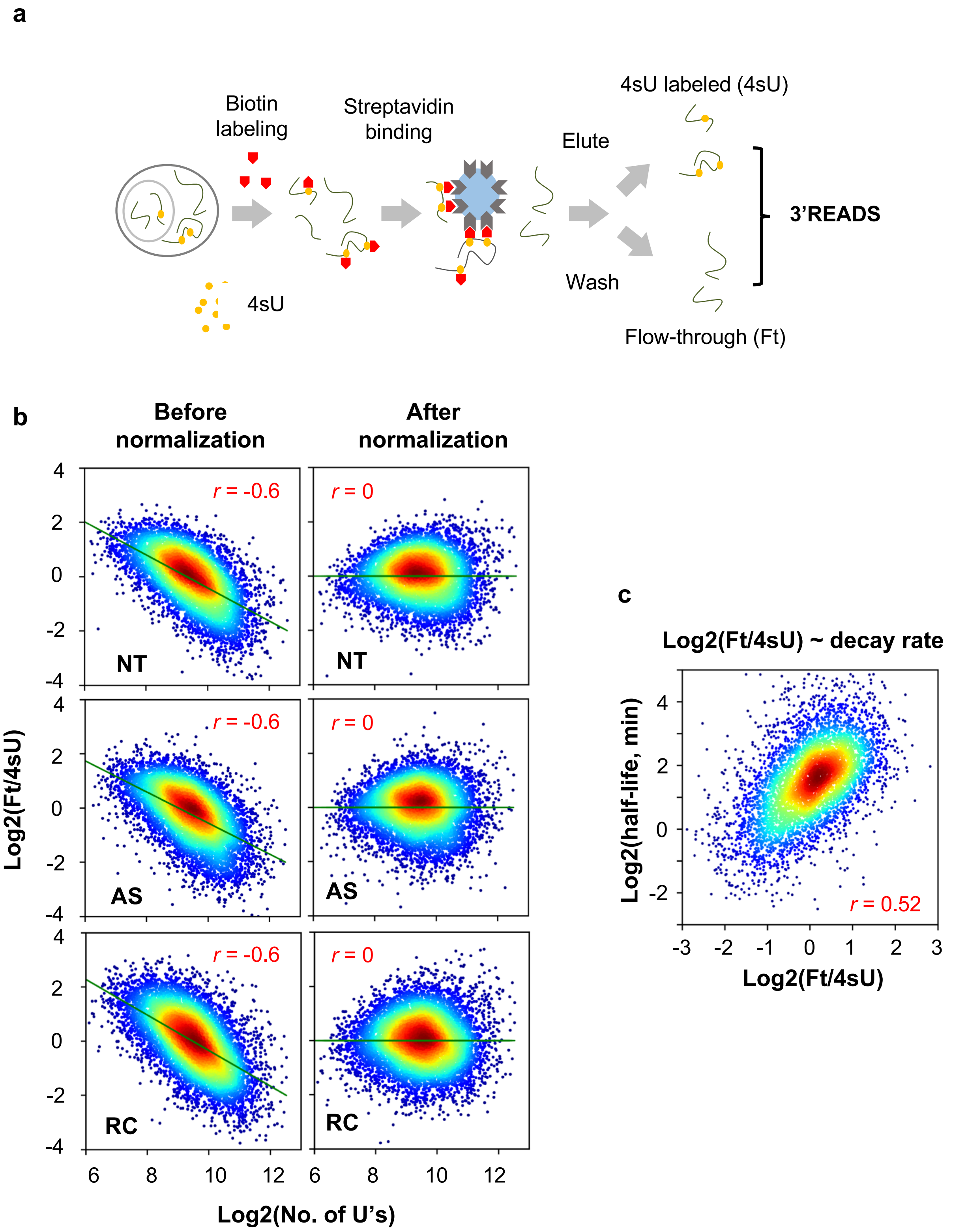

Supplementary Figure 3. RNA Metabolic labeling using 4sU before 3'READS analysis. (a) Schematic of isolation of 4sU-labeled (4sU) and flow-through (Ft) RNAs for 3'READS analysis (see Materials and Methods for detail). **(b)** Normalization of log2(Ft/4sU) values to remove the effect of number of uracils in a transcript (see Materials and Methods for detail). Scatter plots of log2(number of Us per transcript) vs. log2(Ft/4sU) before (top) and after (bottom) normalization are shown. Pearson correlation coefficient (*r*) for each plot is indicated. **(c)** Comparison of log2(Ft/4sU) with log2(half-life). Log2(Ft/4sU) was adjusted in (b). Half-life values (min) were based on a previous study analyzing mRNA decay rate in NIH3T3 cells through transcriptional shutdown by Actinomycin D (see Materials and Methods for detail).

Supplementary Figure 4

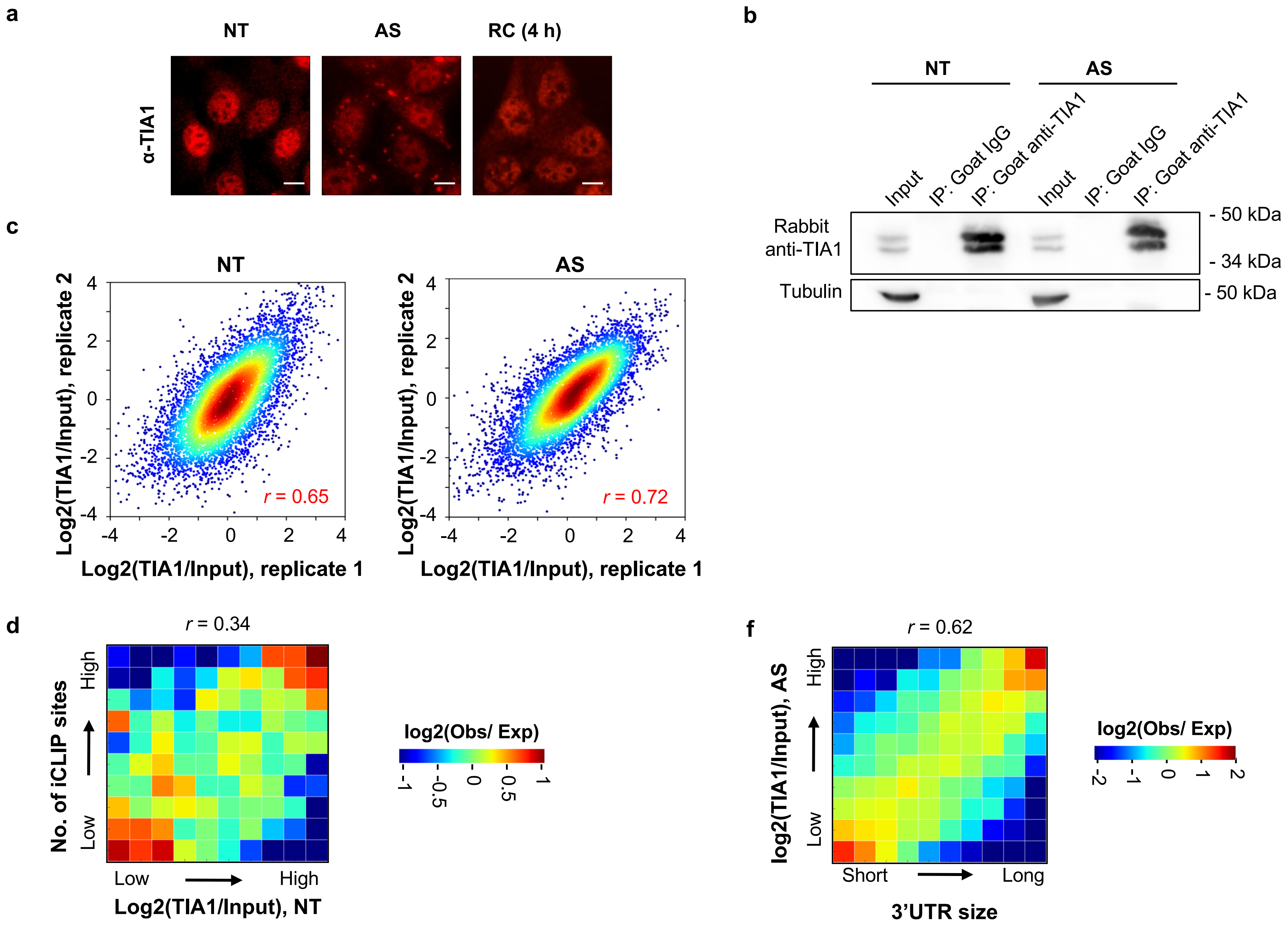

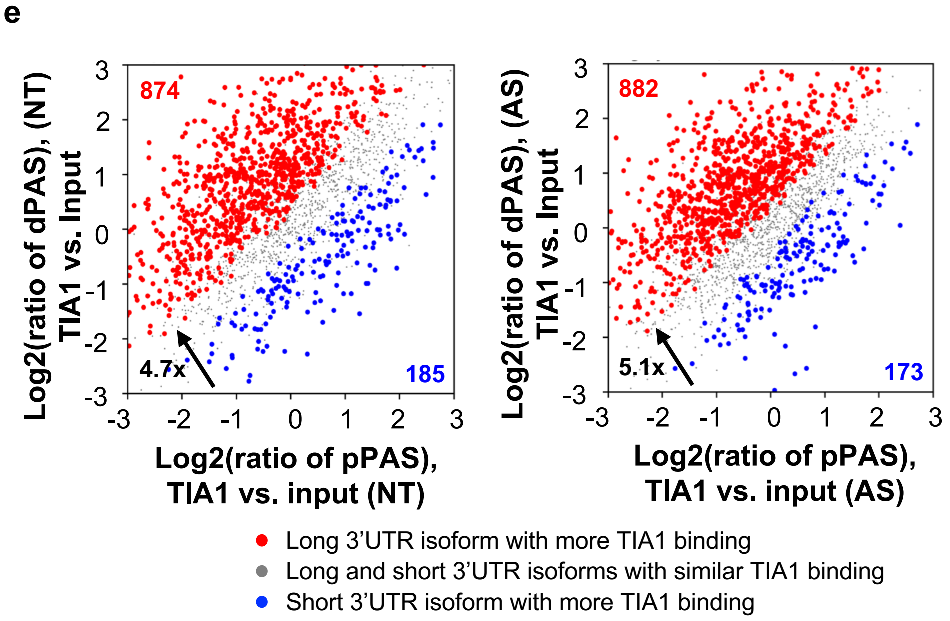

g

Features	r (Pearson)	r (Spearman)	R^2	P-value
3'UTR length	0.62	0.62	0.38	7.5E-31
CDS length	0.47	0.46	0.22	1.2E-04
5'UTR length	0.25	0.27	0.06	1.1E-08
Transcript length	0.73	0.72	0.53	6.8E-69
# ORF exons	0.28	0.28	0.08	1.5E-09
Exon density	-0.26	-0.25	0.07	4.8E-01

Supplementary Figure 4. Analysis of TIA1 interaction with transcripts. (a) Immunocytochemistry analysis of TIA1 in NT, AS (1 h), and RC (4 h) cells. Scale bar: 10 µm. **(b)** Western blot analysis showing the specificity of the goat anti-TIA1 antibody used in the RIP experiments. Normal goat IgG was used as a negative control. **(c)** Scatter plots showing correlation of log2(TIA1/Input) between biological replicates in NT (left) and AS (right) cells. **(d)** Transcript distribution plot showing correlation of TIA1 binding in NT cells and number of reported TIA1 iCLIP sites. Transcripts were evenly divided into ten bins based on number of TIA1 iCLIP sites (y-axis) and log2(TIA1/Input) (x-axis) in NT cells. The number of transcripts in each cell of the 10x10 table was called observed value (Obs) and was divided by the mean of all cells, considered as expected value (Exp). The log2(Obs/Exp) is presented in a heatmap, according to the color scale shown in the graph, which illustrates enrichment or depletion of transcripts in each cell. **(e)** Scatter plots showing log2(ratio) of transcript abundance (RPM) in the TIA1 RIP sample to the Input sample for pPAS isoforms (x-axis) and dPAS isoforms (y-axis) in NT (left) and AS (right) cells. Genes with significant log2(TIA1/Input) difference between two 3'UTR isoforms ($P < 0.05$, Fisher's exact test) in two biological replicates are highlighted in blue (short 3'UTR isoform having more TIA1 binding) or red (long 3'UTR isoform having more TIA1 binding). The numbers of blue and red genes and their ratio are indicated. Genes whose 3'UTR isoforms were not significantly different in TIA1 binding are shown as grey dots. **(f)** Transcript distribution plot showing distribution of transcripts with different 3'UTR sizes (x-axis) and log2(TIA1/Input) values (y-axis) in AS cells. The plot was generated similarly to that in (d). **(g)** Analysis of contributions of various transcript features to log2(TIA1/Input) by linear regression models. Pearson and Spearman coefficient values are shown. R^2 and p-value for linear regression are indicated.

Supplementary Figure 5

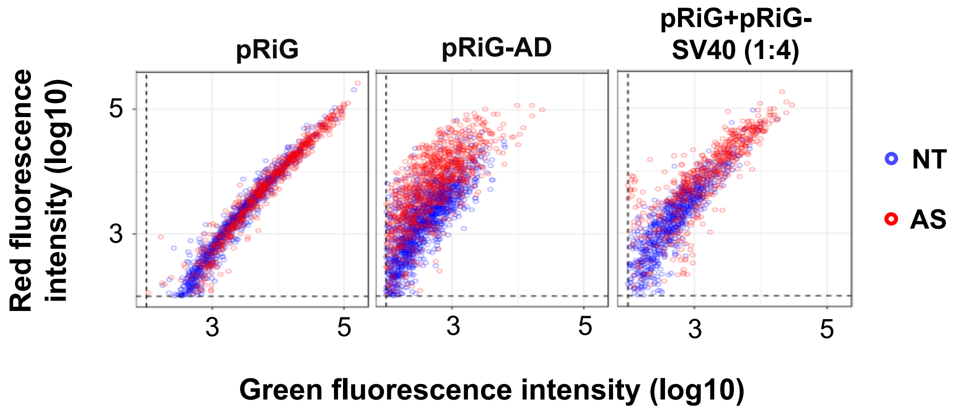

Supplementary Figure 5. FACS analysis of cells transfected with indicated plasmids. pRiG+pRiG-SV40 is a mixture of the two indicated plasmids. Each circle represents one cell, either AS-treated (AS, red) or not treated (NT, blue).

Supplementary Figure 6

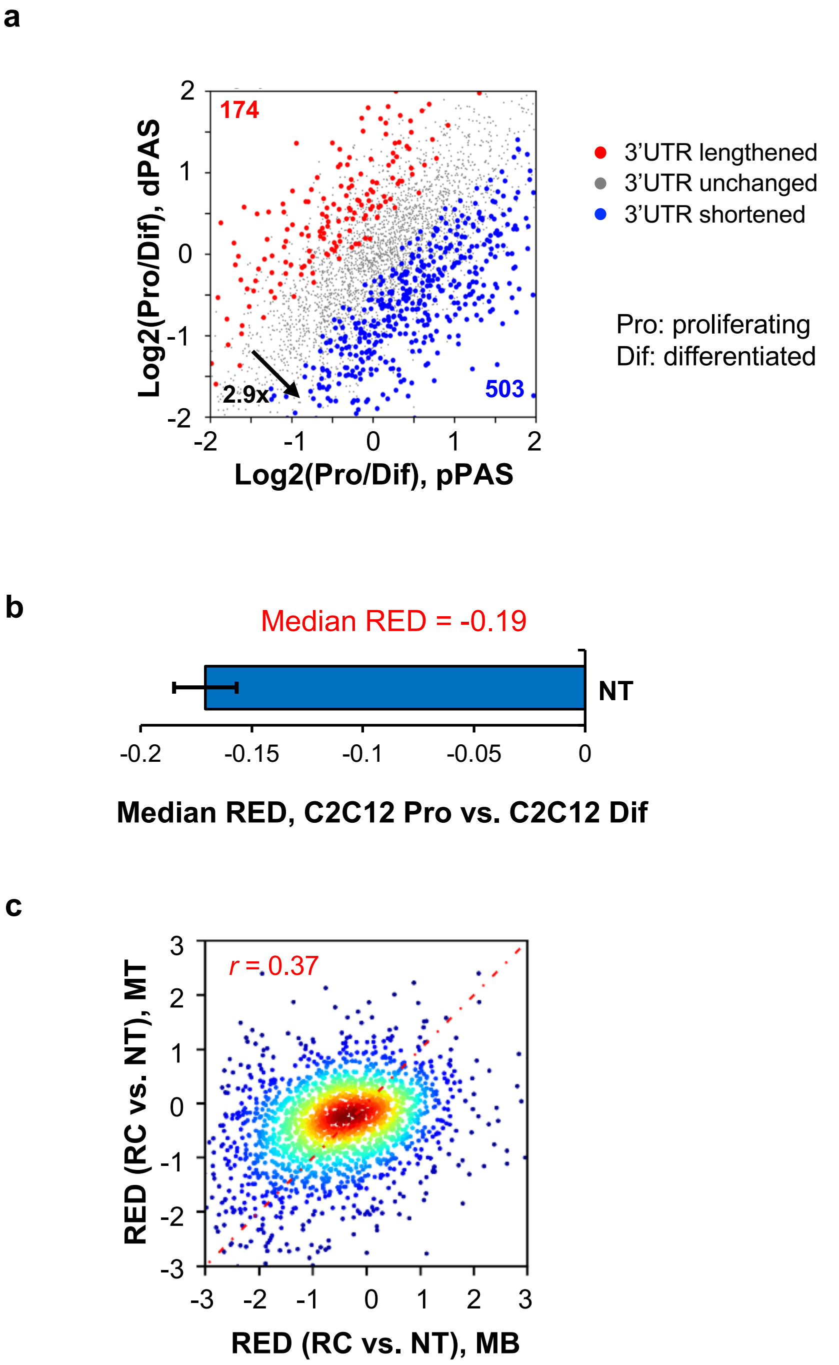

Supplementary Figure 6. 3'UTR APA changes in C2C12 differentiation and stress. (a) Scatter plot showing log2(ratio) of pPAS isoform between proliferating myoblast (MB) and differentiated mytotube (MT) cells (x-axis) vs. that of dPAS isoform (y-axis). Genes with significant difference between dPAS and pPAS isoforms ($P < 0.05$, Fisher's exact test) based on two biological replicates are highlighted in blue (pPAS > dPAS, 3'UTR shortened) or red (pPAS < dPAS, 3'UTR lengthened). Their numbers and the ratio are indicated. Grey dots are genes whose 3'UTR isoforms were not significantly regulated (relative abundance) between MB and MT cells. **(b)** Median RED (mRED) value reflecting 3'UTR size difference between MB and MT cells. Error bar is based on random sampling of data for 20 times (see Materials and Methods for detail). **(c)** RED comparison between MB and MT cells recovering from stress (RC). RED is based on comparison of RC and NT samples.

Supplementary Figure 7

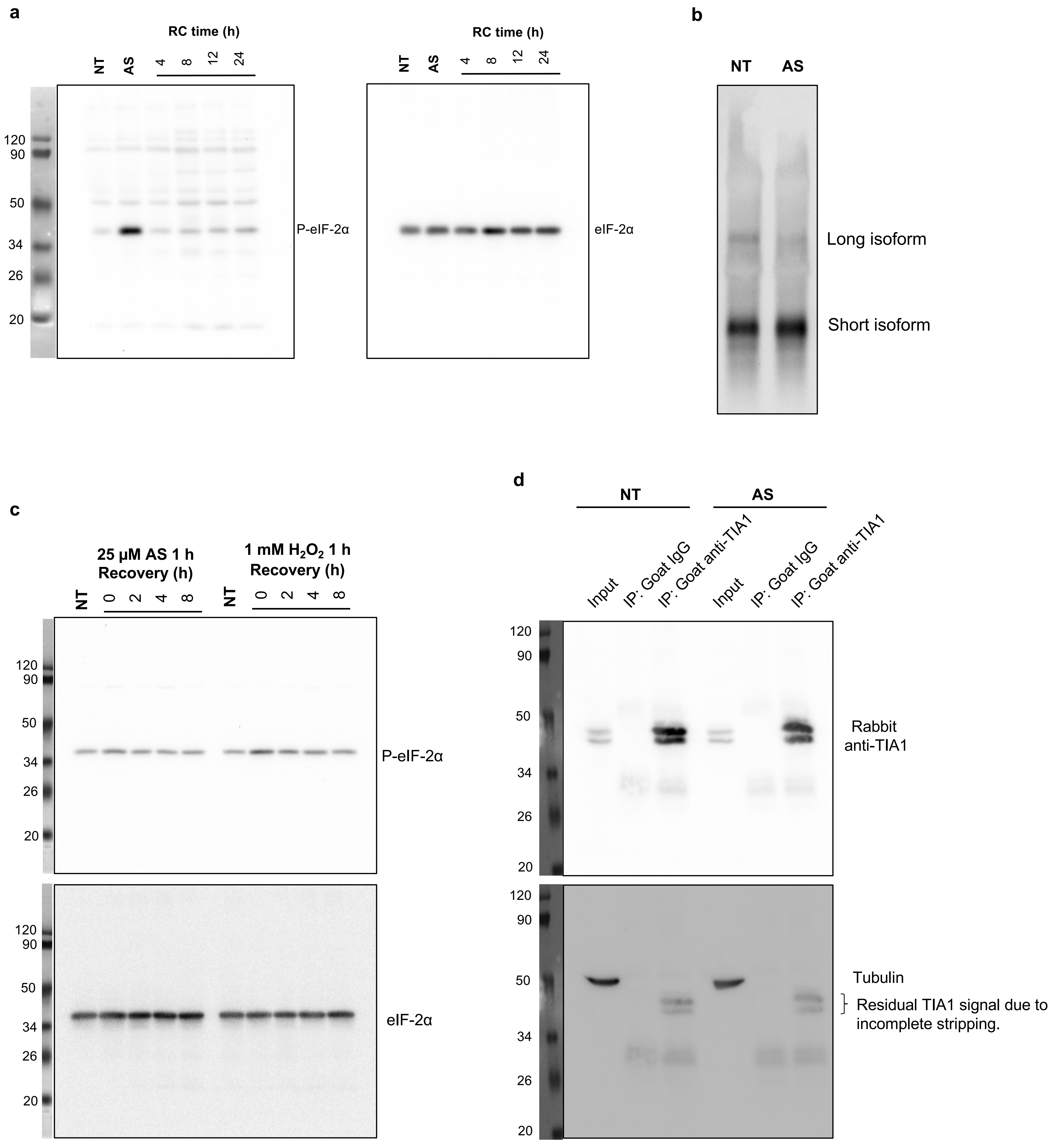

Supplementary Figure 7. Uncropped blot images. (a) Uncropped blot images for Figure 1b. **(b)** Uncropped blot images for Figure 6c. **(c)** Uncropped blot images for Supplementary Figures 2a and 2c. **(d)** Uncropped blot images for Supplementary Figure 4b. Pre-stained molecular weight markers were imaged using white light.
